# Causal relationships between gut microbrome and digestive system diseases: A two-sample Mendelian randomization study

**DOI:** 10.1097/MD.0000000000037735

**Published:** 2024-04-26

**Authors:** Wenjing Ding, Liangliang Chen, Jianguo Xia, Gang Dong, Biao Song, Bei Pei, Xuejun Li

**Affiliations:** a The Second Clinical Medical School, Anhui University of Chinese Medicine, Hefei, Anhui, China; b Department of Gastroenterology, The Second Affiliated Hospital of Anhui University of Chinese Medicine, Hefei, Anhui, China.

**Keywords:** causal relationships, digestive system diseases, gut microbiota, Mendelian randomization

## Abstract

Growing evidences of recent studies have shown that gut microbrome are causally related to digestive system diseases (DSDs). However, causal relationships between the gut microbiota and the risk of DSDs still remain unclear. We utilized identified gut microbiota based on class, family, genus, order and phylum information and digestive system diseases genome-wide association study (GWAS) dataset for two-sample Mendelian randomization (MR) analysis. The inverse variance weighted (IVW) method was used to evaluate causal relationships between gut microbiota and 7 DSDs, including chronic gastritis, colorectal cancer, Crohn’s disease, gastric cancer, gastric ulcer, irritable bowel syndrome and esophageal cancer. Finally, we verified the robustness of MR results based on heterogeneity and pleiotropy analysis. We discovered 15 causal associations with genetic liabilities in the gut microbiota and DSDs, such as *genus Victivallis, genus RuminococcaceaeUCG*005, *genus Ruminococcusgauvreauiigroup, genus Oxalobacter* and so on. Our MR analysis revealed that the gut microbiota is causally associated with DSDs. Further researches of the gut microbiota and the pathogenesis of DSDs are still significant and provide new methods for the prevention and treatment of DSDs.

## 1. Introduction

Digestive system diseases (DSDs) are the most common disorders globally, including esophageal and gastrointestinal diseases (chronic gastritis, colorectal cancer, Crohn’s disease, gastric cancer, gastric ulcer, irritable bowel syndrome and esophageal cancer). Chronic gastritis is persistent inflammatory lesions in the gastric mucosa and it has an influence on above half of the global population in various degrees.^[1,2]^ For colorectal cancer, it is the second most common cause of death from malignant tumor.^[3]^ Risk factors about colorectal cancer consist of family and personal medical history (such as family genetics and history, Crohn’s Disease and so on) and lifestyle (such as patterns of daily dietary and the habit of cigarette smoking).^[4]^ Gastric cancer is the fifth most common cancer with approximately 784,000 deaths all over the world in 2018 and one of the most risk factors of gastric cancer is *Helicobacter pylori* infection.^[5]^ The study in^[6]^ illustrated that gastric ulcer is a disability in the stomach wall penetrating through the entire mucosa and the muscularis mucosae deeply and it can be also caused by toxic factor of *H. pylori* infection. Irritable bowel syndrome^[7]^ is one of the most common gut–brain interaction disorders and it has an influence on above one tenth people worldwide with including abdominal pain related to a change in stool form or frequency.^[8]^ For esophageal cancer, it is the seventh most common cancer globally and it leads to around 450,000 patient deaths each year.^[9]^ Risk factors about esophageal cancer are tobacco smoking, alcohol overconsumption, intake of red meat and the consumption of very hot beverages.^[10]^ Of note, some evidences had found out that the gut microbiota might have a causal link with the development of digestive system disorders.^[11]^ Gut microbiota located in the gastrointestinal tract contains thousands of bacterial species and trillions of microorganisms and it play an important role in a variety of diseases.^[12]^ Its inflammation might have a causal association with the initiation, development and progression of digestive cancer.^[13,14]^ Accumulating evidence demonstrated that specific gut bacteria, which are considered as interbacterial communication, are related to the development of gastrointestinal cancers.^[15]^ In,^[16]^ authors have reported that differences in microbial composition have been linked to chronic digestive diseases such as inflammatory bowel disease and colorectal cancer. The study in^[17]^ investigated the significance of a diminution in gut microbial diversity for host metabolism. The research has demonstrated that the gut microbiota is related to the development of colorectal cancer.^[18]^ Although some studies have demonstrated causal relationships between gut microbiota and DSDs, the detailed development of DSDs still remains unclear. Hence, subsequent researches play a crucial role in the exploration of causal links between gut microbiota and DSDs.

Mendelian randomization (MR) is a well-known technique for the assessment of causal relationships between exposure dataset and outcome dataset by using genome-wide association study (GWAS) summary data. In our context, the MR statistical analysis was employed to explore causal associations between gut microbiota as exposure and DSDs as outcome dataset and showcased results based on forest plots.

## 2. Materials and methods

### 2.1. Ethical statement

**Figure 1.**
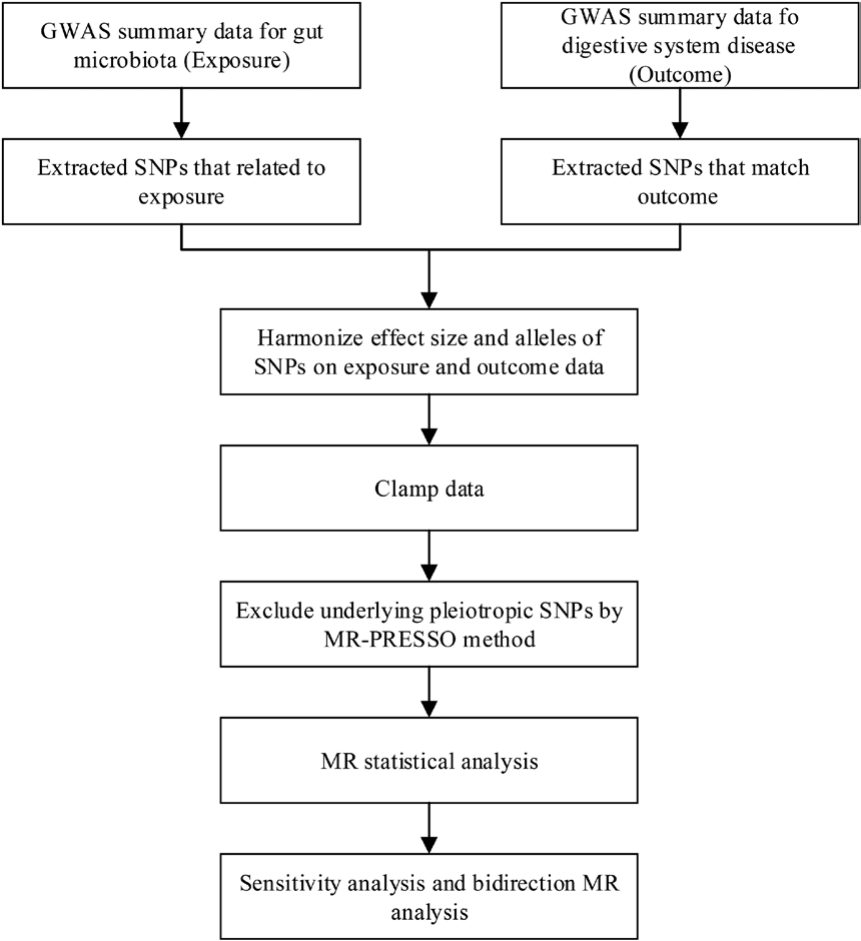
The flowchart of our study. GWAS = genome-wide association study, MR = Mendelian randomization, SNP = single nucleotide polymorphism.

All of dataset employed in our study were large-scale public GWAS summary data. Ethical approval and consent to participate were acquired in all original studies. The flowchart of the process is illustrated in Figure 1. In a nutshell, the human gut microbiota was considered as exposure dataset and DSDs were considered as outcome dataset.

### 2.2. Gut microbiota exposure

Human gut microbiota GWAS summary data were selected from the MiBioGen consortium (https://mibiogen.gcc.rug.nl/) with 16S ribosomal RNA gene sequencing profiles and genotyping data from 18,340 sample sizes. The gut microbiota dataset contains 211 taxa, 131 genera, 35 families, 20 orders, 16 classes and 9 phyla.^[19]^ In our study, we selected a series of parameters to make sure of the accuracy of results. The selection criteria of instrumental variables (IVs) from single nucleotide polymorphisms (SNPs) are as followed^[20,21]^: the genome-wide statistical significance threshold is less than 1 × 10^−6^ as candidate IVs; the linkage disequilibrium threshold was set as R^2^ < 0.01 and distance was set as kb = 10000 kb to avoid linkage disequilibrium; F-statistics^[22]^ of each IV was calculated by F = R^2^ × (N − 2)/(1 − R^2^), where R^2^ represents the genetic variant explanation of the exposure variance and N represents sample sizes. Meanwhile, F > 10 indicated a strong instrument and was retained.^[23]^

### 2.3. Digestive system diseases outcome

The summary data of 7 DSDs were derived from GWAS (portal: https://gwas.mrcieu.ac.uk/), including chronic gastritis (N = 361,194, ncase = 1790, ncontrol = 359,404), colorectal cancer (N = 470,002, ncase = 6581, ncontrol = 463,421), Crohn’s disease (N = 40,266, ncase = 12,194, ncontrol = 28,072), gastric cancer (N = 476,116, ncase = 1029, ncontrol = 475,087), gastric ulcer (N = 474,278, ncase = 6293, ncontrol = 467,985), irritable bowel syndrome (N = 486,601, ncase = 53,400, ncontrol = 433,201) and esophageal cancer (N = 372,756, ncase = 740, ncontrol = 372,016). The outcome detailed characteristic of GWAS were illustrated in Table 1.

**Table 1:** Detailed characteristics of GWAS related to outcome dataset in the study.

Type	Population	Sample size	Number of SNPs	ID in GWAS
Chronic gastritis	European	361,194	10,262,134	ukb-d-K11_CHRONGASTR
Colorectal cancer	European	470,002	24,182,361	ebi-a-GCST90018808
Crohn’s disease	Mixed	40,266	9457,998	ebi-a-GCST004132
Gastric cancer	European	476,116	24,188,662	ebi-a-GCST90018849
Gastric ulcer	European	474,278	24,178,780	ebi-a-GCST90018851
Irritable bowel syndrome	European	486,601	9739,966	ebi-a-GCST90016564
Oesophageal cancer	European	372,756	8970,465	ieu-b-4960

### 2.4. Mendelian randomization analysis

We performed a MR analysis to explore causal relationships between gut microbiota and DSDs. MR statistical analysis should comply with 3 core assumptions to reduce estimate bias.^[24]^ Initially, genetic variants must be associated with gut microbiota exposure dataset. Subsequently, IVs of gut microbiota exposure dataset are supposed to be uncorrelated with confounder, which are linked to both gut microbiota and DSDs. Lastly, IVs can only impact DSDs through gut microbiota to avoid horizontal pleiotropy. If a particular taxon had 1 SNP as an IV, Wald ratio method was leveraged for MR analysis. If taxon had more than 1 SNP as IVs, 4 MR regression methods were utilized, such as inverse variance weighted (IVW),^[25]^ weighted median,^[26]^ weighted mode^[27]^ and MR-Egger.^[28]^ IVW method was considered as the primary analysis (*P* < .05) and other regression methods were served as complements. Meanwhile, the Benjamini-Hochberg method was used to adjust *P* value and the corrected p_FDR value was displayed in tables in Section 3. In order to evaluate the sensitive of our MR analysis,^[29]^ Cochrane’Q test^[30]^ was utilized to obtain heterogeneity. For horizontal pleiotropy, MR-Egger and MR-PRESSO global test were employed and there were no evidence of both heterogeneity and horizontal pleiotropy based on all of *P* > .05. Finally, the leave-one-out method was leveraged to investigate the reliability of harmonizing both exposure and outcome dataset.

In our paper, all MR statistical analyses were implemented using “TwoSampleMR” (v0.5.7)^[31]^ and “MR-PRESSO” (v 1.0)^[32]^ packages in R v 4.3.1. In order to accelerate our statistical analysis, “doParallel” (v 1.0.17) package was employed.

## 3. Results

### 3.1. Selection of instrumental variables for gut microbiome

After calculating set parameters, 302 bacterial characteristics with 5 biological levels (class, family, genus, order and phylum) were selected. The F-statistics of IVs ranged from 20,631 to 88,430, thereby all of selected IVs were remarkable >10, which manifested the absence of weak instrument bias.

### 3.2. Results of Mendelian randomization analysis

#### 3.2.1. Causal relationships between gut microbiome and chronic gastritis

**Figure 2.**
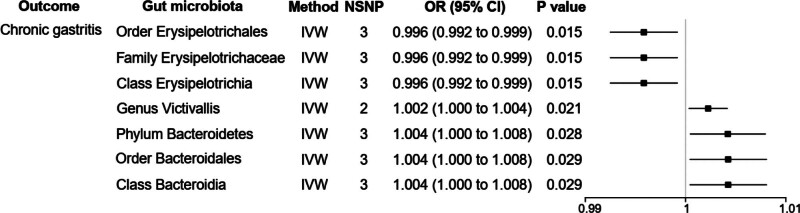
MR results of causal relationships between gut microbiome and chronic gastritis (*P* < 1 × 10^−6^). MR = Mendelian randomization.

According to results of the IVW method, *order Erysipelotrichales* [OR = 0.996, 95% CI: 0.992–0.999, *P *= .015], *family Erysipelotrichaceae* [OR = 0.996, 95% CI: 0.992–0.999, *P *= .015] and *class Erysipelotrichia* [OR = 0.996, 95% CI: 0.992–0.999, *P *= .015] had a decreased evidence of chronic gastritis. Otherwise, *genus Victivallis* [OR = 1.002, 95% CI: 1.000–1.004, *P *= .021], *phylum Bacteroidetes* [OR = 1.004, 95% CI:1.000–1.008, *P *= .028], *order Bacteroidales* [OR = 1.004, 95% CI: 1.000–1.008, *P* = .029], as well as *class Bacteroidia* [OR = 1.004, 95% CI: 1.000–1.008, *P *= .029] had a positive evidence of chronic gastritis in Figure 2.

#### 3.2.2. Causal relationships between gut microbiome and colorectal cancer

**Figure 3.**
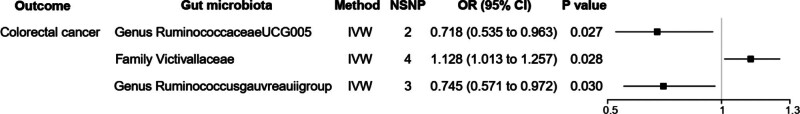
MR results of causal relationships between gut microbiome and colorectal cancer (*P* < 1 × 10^−6^). MR = Mendelian randomization.

Using the IVW method, *genus RuminococcaceaeUCG*005 [OR = 0.718, 95% CI: 0.535–0.963, *P *= .027] and *genus Ruminococcusgauvreauiigroup* [OR = 0.745, 95% CI: 0.571–0.972, *P *= .030] were negative related with the risk of colorectal cancer. On the contrary, *family Victivallaceae* [OR = 1.128, 95% CI: 1.013–1.257, *P *= .028] were positive related with the risk of colorectal cancer in Figure 3.

#### 3.2.3. Causal relationships between gut microbiome and Crohn’s disease

**Figure 4.**
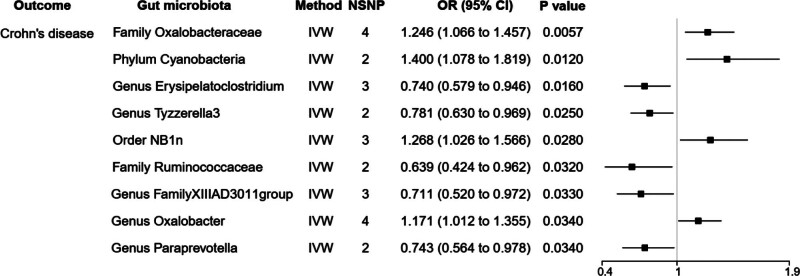
MR results of causal relationships between gut microbiome and Crohn’s disease (*P < *1 *× *10^*−*6^). MR = Mendelian randomization.

Based on the IVW method in Figure 4, *family Oxalobacteraceae* [OR = 1.246, 95% CI: 1.066–1.457, *P *= .0057], *phylum Cyanobacteria* [OR = 1.400, 95% CI: 1.078–1.819, *P *= .012], *order NB*1*n* [OR = 1.268, 95% CI: 1.026–1.566, *P *= .028], *genus Oxalobacter* [OR = 1.171, 95% CI: 1.012–1.355, *P *= .034] had a great risk of Crohn’s disease. *Genus Erysipelatoclostridium* [OR = 0.740, 95%CI:0.579–0.946, *P *= .016], *genus Tyzzerella*3 [OR = 0.781, 95% CI: 0.630–0.969, *P *= .025], *family Ruminococcaceae* [OR = 0.639, 95% CI: 0.424–0.962, *P *= .032], *genus FamilyXIIIAD*3011*group* [OR = 0.711, 95% CI: 0.520–0.972, *P *= .033] and *genus Paraprevotella* [OR = 0.743, 95% CI: 0.564–0.978, *P *= .033] acted as preventive results of Crohn’s disease.

#### 3.2.4. Causal relationships between gut microbiome and gastric cancer

**Figure 5.**
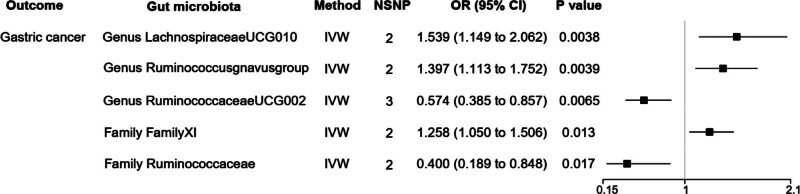
MR results of causal relationships between gut microbiome and gastric cancer (*P* < 1 × 10^−6^). MR = Mendelian randomization.

When identifying gut microbiome with gastric cancer, we found that *genus Ruminococ caceaeUCG*002 [OR = 0.574, 95% CI: 0.385–0.857, *P *= .0065] and *family Ruminococcaceae* [OR = 0.400, 95% CI: 0.189–0.848, *P *= .017] reduced the risk of gastric cancer. On the other hand, *genus LachnospiraceaeUCG*010 [OR = 1.539, 95% CI: 1.149–2.062, *P *= .0038], *genus Ruminococcusgnavusgroup* [OR = 1.397, 95% CI: 1.113–1.752, *P *= .0039] as well as *family FamilyXI* [OR = 1.258, 95% CI: 1.050–1.506, *P *= .013] increased the risk of gastric cancer in Figure 5.

#### 3.2.5. Causal relationships between gut microbiome and gastric ulcer

**Figure 6.**
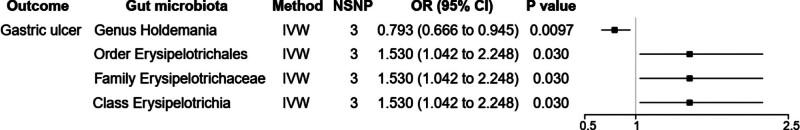
MR results of causal relationships between gut microbiome and gastric ulcer (*P* < 1 × 10^−6^). MR = Mendelian randomization.

The results provided in Figure 6 from the IVW method showed that *genus Holdemania* [OR = 0.793, 95% CI: 0.666–0.945, *P *= .0097] had a decreased incidence of gastric ulcer. Alternatively, *order Erysipelotrichales* [OR = 1.530, 95% CI: 1.042–2.248, *P *= .030], *family Erysipelotrichaceae* [OR = 1.530, 95% CI: 1.042–2.248, *P *= .030] and *class Erysipelotrichia* [OR = 1.530, 95% CI: 1.042–2.248, *P *= .030] had an increased incidence of gastric ulcer.

#### 3.2.6. Causal relationships between gut microbiome and irritable bowel syndrome

**Figure 7.**
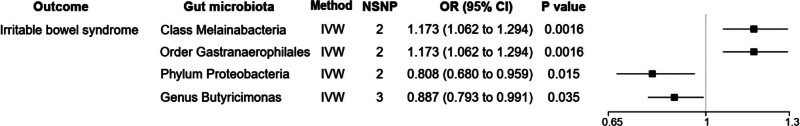
MR results of causal relationships between gut microbiome and irritable bowel syndrome (*P* < 1 × 10^−6^). MR = Mendelian randomization.

When determining gut microbiome with irritable bowel syndrome in Figure 7, we demonstrated that *phylum Proteobacteria* [OR = 0.808, 95% CI: 0.680–0.959, *P *= .015] and *genus Butyricimonas* [OR = 0.887, 95% CI: 0.793–0.991, *P *= .035] were linked to a low risk of irritable bowel syndrome. *Class Melainabacteria* [OR = 1.173, 95% CI: 1.062–1.294, *P *= .0016] and *order Gastranaerophilales* [OR = 1.173, 95% CI: 1.062–1.294, *P *= .0016] were linked to a high risk of irritable bowel syndrome.

#### 3.2.7. Causal relationships between gut microbiome and esophageal cancer

**Figure 8.**
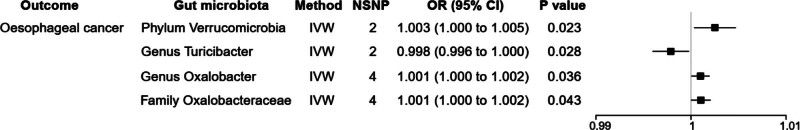
MR results of causal relationships between gut microbiome and esophageal cancer (*P < *1 *× *10^*−*6^). MR = Mendelian randomization.

Figure 8 illustrated that *genus Turicibacter* [OR = 0.998, 95% CI: 0.996–1.000, *P *= .028] reduced the risk of esophageal cancer. Conversely, *phylum Verrucomicrobia* [OR = 1.003, 95% CI: 1.000–1.005, *P *= .023], *genus Oxalobacter* [OR = 1.001, 95% CI: 1.000–1.002, *P *= .036] and *family Oxalobacteraceae* [OR = 1.001, 95% CI: 1.000–1.002, *P *= .043] were positive associated with the risk of esophageal cancer.

### 3.3. Sensitivity analysis

The robustness of our MR results between gut microbiome and DSDs were evaluated by the sensitive analysis. Significant heterogeneity and horizontal pleiotropy were both identified. Meanwhile, correct causal directions and Steiger *P* value^[29]^ were also identified. As shown in Table 2, we did not find significant heterogeneity and horizontal pleiotropy due to all *P > *.05. For Benjamini-Hochberg corrected method, it is worth noting that none of MR results in our study adapt to a significant level owing to false negatives.^[33]^ In addition, the leave-one-out test demonstrated that excluded any SNP had no impact on our MR estimations.

**Table 2:** MR estimates for causal relationships between gut microbiome and DSDs (*P* < 1 × 10^−6^).

Outcome	Gut microbiota	p_FDR	Correct causal direction	Steiger pval	P heterogeneity	P pleiotropy
Chronic gastritis	***Order Erysipelotrichales***	0.972	TRUE	4.12E–17	0.858	0.919
	***Family Erysipelotrichaceae***	0.972	TRUE	4.12E–17	0.858	0.919
	***Class Erysipelotrichia***	0.972	TRUE	4.12E–17	0.858	0.919
	***Genus Victivallis***	0.972	TRUE	4.96E–12	0.807	NA
	***Phylum Bacteroidetes***	0.972	TRUE	3.25E–18	0.395	0.478
	***Order Bacteroidales***	0.972	TRUE	1.13E–17	0.397	0.478
	***Class Bacteroidia***	0.972	TRUE	1.13E–17	0.397	0.478
Colorectal cancer	***Genus RuminococcaceaeUCG005***	0.775	TRUE	8.14E–19	0.376	NA
	***Family Victivallaceae***	0.775	TRUE	1.47E–23	0.332	0.305
	***Genus Ruminococcusgauvreauiigroup***	0.775	TRUE	1.12E–16	0.365	0.448
Crohn’s disease	***Family Oxalobacteraceae***	0.283	TRUE	1.43E–18	8.66E-01	0.725
	***Phylum Cyanobacteria***	0.283	TRUE	4.81E–09	9.68E-01	NA
	***Genus Erysipelatoclostridium***	0.283	TRUE	9.22E–13	7.48E-01	0.829
	***Genus Tyzzerella3***	0.283	TRUE	5.26E–11	8.68E-01	NA
	***Order NB1n***	0.283	TRUE	1.67E–13	2.95E-01	0.469
	***Family Ruminococcaceae***	0.283	TRUE	3.77E–07	3.66E-01	NA
	***Genus FamilyXIIIAD3011group***	0.283	TRUE	9.96E–14	5.45E-01	0.479
	***Genus Oxalobacter***	0.283	TRUE	2.66E–20	5.53E-01	0.319
	***Genus Paraprevotella***	0.283	TRUE	9.53E–09	5.33E-01	NA
Gastric cancer	***Genus LachnospiraceaeUCG010***	0.150	TRUE	1.06E–10	0.525	NA
	***Genus Ruminococcusgnavusgroup***	0.150	TRUE	3.10E–12	0.450	NA
	***Genus RuminococcaceaeUCG002***	0.168	TRUE	4.85E–21	0.347	0.478
	***Family FamilyXI***	0.245	TRUE	8.11E–12	0.405	NA
	***Family Ruminococcaceae***	0.260	TRUE	6.96E–12	0.546	NA
Gastric ulcer	***Genus Holdemania***	0.581	TRUE	5.94E–17	0.897	0.729
	***Order Erysipelotrichales***	0.581	TRUE	1.54E–15	0.186	0.454
	***Family Erysipelotrichaceae***	0.581	TRUE	1.54E–15	0.186	0.454
	***Class Erysipelotrichia***	0.581	TRUE	1.54E–15	0.186	0.454
Irritable bowel syndrome	***Class Melainabacteri***	0.060	TRUE	2.37E–12	0.859	NA
	***Order Gastranaerophilales***	0.060	TRUE	2.49E–12	0.844	NA
	***Phylum Proteobacteria***	0.386	TRUE	3.14E–11	0.857	NA
	***Genus Butyricimonas***	0.610	TRUE	8.23E–22	0.874	0.791
Oesophageal cancer	***Phylum Verrucomicrobia***	0.726	TRUE	5.74E–11	0.636	NA
	***Genus Turicibacter***	0.726	TRUE	5.88E–12	0.436	NA
	***Genus Oxalobacter***	0.726	TRUE	8.82E–25	0.769	0.783
	***Family Oxalobacteraceae***	0.726	TRUE	1.16E–23	0.726	0.846

## 4. Discussion

In our context, it is the first MR study to explore causal relationships between gut microbiota and DSDs (chronic gastritis, colorectal cancer, Crohn’s disease, gastric cancer, gastric ulcer, irritable bowel syndrome and esophageal cancer). We demonstrated that there were abundant evidences indicating that specific gut microbiota play a vital role in the development of DSDs. For example, *order Erysipelotrichales* was causally associated with chronic gastritis and *genus RuminococcaceaeUCG*005 was causally related to colorectal cancer. As shown in our MR results, statistical analysis have suggested that *order Erysipelotric hales* and *family Erysipelotrichaceae* are increased risk factors for chronic gastric but are decreased risk factors for gastric ulcer. For *genus RuminococcaceaeUCG*005 and *Genus RuminococcaceaeUCG*002, both of them were associated with an increased risk of colorectal cancer and colorectal cancer, respectively. In addition, *family Oxalobacteraceae* and *genus Oxalobacter* both participated in the link between Crohn’s disease and esophageal cancer. These findings provided new evidences for further cognitive and treatment of DSDs.

Recently, there were lots of literature to investigate causal relationships between gut microbiota and DSDs. In,^[34]^ authors have reported that *genus RuminococcaceaeUCG*002 was related to increase gastric cancer, but our results showcased the negative risk of gastric cancer in Table 1. *Ruminococcus* is a main member of the human gut microbiota that plays a significant role in digestion based on host enzymes.^[35]^ Hence, families of *Ruminococcus*, including *genus Ruminococcusgauvreauiigroup* and *genus Ruminococcusgnavusgroup*, were concerned with DSDs in our MR results. Meanwhile, *Ruminococcusgnavus* played a crucial role in the development of Crohn’s diseas.^[36]^ The study in^[37]^ suggested that *Bacteroidetes* reduction can lead to inflammatory cytokines (IL-6), which may damage DNA and cause chronic inflammation and inflammation-associated cancers.^[38]^ In another research,^[39]^ authors obtained findings that genus *ErysipelotrichaceaeUCG*004 and the lower profusion of *Erysipelotrichaceae* were evaluated correlated with *H. pylori* infection and can cause chronic gastritis and gastric ulcer, which supported our MR results. Sufficient studies manifested that genera/species *Lachnospiraceae* increased a positive risk of gastric cancer in various counties, such as China, South Korea and Mexico.^[40–42]^ Furthermore, an enriched level of *Proteobacteria* was related to the development of colorectal cancer.^[43]^ Previous MR studies have revealed causal links between gut microbiota and autoimmune diseases (such as inflammatory bowel disease, type 1 diabetes),^[44]^ blood metabolites,^[45]^ 8 types of cancers (including colorectal cancer and gastric cancer),^[46]^ colorectal cancer^[47]^ and breast cancer.^[33]^ For DSDs, the study in^[47]^ detected *family Verrucomicrobiaceae, family Enterobacteriaceae, genus Akkermansia, genus Blautia* and *genus Ruminococcus* had association with the risk of colorectal cancer. Besides, the study in^[46]^ observed a causal relationship between *family Peptostreptococcaceae* and gastric cancer. Meanwhile, *genus Tyzzerella*3, *genus Ruminococcustorquesgroup, order Verrucomicrobiales, class Verrucomicrobiae*, et al were causally associated with colorectal cancer.

Additionally, a clinical study has found out that gut microbiota of some taxa, called *Bacteroides, Fusobacterium* and *Prevotella*, revealed vital correlations with differentially expressed metabolites between *H. pylori* positive and *H. pylori* negative individuals with chronic gastritis.^[48]^ Meanwhile, a recent study suggested that chronic atrophic gastritis (CAG) patients with Hp-II infected occupied high abundances of several dominant microbiota (e.g. *Neisseria, Staphylococcus* and *Haemophilus*).^[49]^ For colorectal cancer, a mice experiment has reported that gut microbiota *campylobacter jejuni* can generate a genotoxin with DNase activity to provoke DNA double-strand breaks by directly, which can cause the paroxysm of CC.^[50]^ In,^[51]^ the characteristics of microbiota in 276 patients with GC was obsessed that the cancerous, paracancerous and normal tissues took over similar main bacteria, for *H. pylori, Halomonas*, and *Shewanella* in paracancerous tissues, while *Streptococcus, Selenomonas, Fusobacterium, Propionibacterium*, and *Corynebacterium* were increased in cancerous parts. A systematic case-control study have documented that family *Enterobacteriaceae* (phylum *Proteobacteria*), family *Lactobacillaceae* and genus *Bacteroides* were increased in patients with IBS based on MEDLINE, EMBASE, Cochrane CDSR and CENTRAL databases, which is the potentially harmful gut microbiota.^[52]^

Our research has following advantages. First, our MR analysis is the first research to explore how gut microbiota and the risk of DSDs interact. Second, grant quantities of publicly open GWAS dataset are ensured reliably and effectively. Finally, the time spent on this MR analysis was very cost-effective for outcome sources we selected, in opposition to the time-consuming randomized controlled trials (RCTs). Moreover, our studies still had some inescapable shortcomings that should be taken into account. First, the majority of sample in GWAS summary data were European due to geographical constraints, which may give rise to bias estimates due to constraints of GWAS dataset. Second, only one statistical significance threshold parameter (1 *× *10^*−*6^) was chosen, so it may lead to loss of genetic liabilities of gut microbiota. Finally, environment and the dietary habit can affect the gut microbiota profoundly, thus more relevant confounder are supposed to be deemed. Above studies indicated that future studies are supposed to utilize a comprehensive method to make sense of the pathogenesis of digestive system disorders between gut microbiota and genes/environment.

## 5. Conclusions

In conclusion, our MR analysis revealed causal relationships between gut microbiota and DSDs. Our results reported that there are 4 increased causal directions and 3 decreased causal directions with chronic gastritis, 2 increased causal directions and 1 decreased causal direction with colorectal cancer, 4 increased causal directions and 5 decreased causal directions with Crohn’s disease, 3 increased causal directions and 2 decreased causal directions with gastric cancer, 3 increased causal directions and 1 decreased causal direction with gastric ulcer, 2 increased causal directions and 2 decreased causal directions with irritable bowel syndrome, as well as 3 increased causal directions and 1 decreased causal direction with esophageal cancer. Subsequent studies should further reveal potential mechanisms by which the gut microbiota deeply affects DSDs.

## Acknowledgments

The data used in this study were generously provided by the MiBioGen and GWAS repository. We extend our heartfelt gratitude for their invaluable contributions to this research and for the active participation of the study participants

## Author contributions

**Conceptualization:** Wenjing Ding, Liangliang Chen, Jianguo Xia, Gang Dong, Xuejun Li.

**Funding acquisition:** Xuejun Li.

**Investigation:** Wenjing Ding, Liangliang Chen, Biao Song, Bei Pei.

**Methodology:** Wenjing Ding.

**Visualization:** Wenjing Ding.

**Writing – original draft:** Wenjing Ding.

**Writing – review & editing:** Liangliang Chen, Jianguo Xia, Gang Dong, Biao Song, Bei Pei, Xuejun Li.
